# Asymptomatic Systemic Mastocytosis Uncovered During Routine Colonoscopy: A Case Report

**DOI:** 10.7759/cureus.60286

**Published:** 2024-05-14

**Authors:** Amina Sara Matmatte, Dharaneswari Hari Narayanan, Twan Sia, Saad Shami, Jerry Fu, Puay Eng Tan, John Leung

**Affiliations:** 1 Gastroenterology, Allergy, and Immunology, Boston Specialists, Boston, USA; 2 Internal Medicine, Stanford University School of Medicine, Stanford, USA; 3 Department of Pathology, Steward Carney Hospital, Dorchester, USA

**Keywords:** indolent, occult presentations, colonoscopy, asymptomatic, systemic mastocytosis

## Abstract

We report an atypical case of systemic mastocytosis in a 66-year-old asymptomatic female, diagnosed incidentally during a routine colonoscopy. This case highlights the diversity of clinical presentations and emphasizes the role of colonoscopy and the need for thorough histopathological examinations in routine endoscopic procedures with subtle abnormalities.

## Introduction

Systemic mastocytosis (SM) refers to a heterogeneous group of disorders characterized by abnormal accumulation and proliferation of mast cells in one or more organ systems [1]. SM disorders range from extremely aggressive to benign, underscoring the importance of early detection. Per World Health Organization (WHO) criteria, diagnosis of SM requires meeting one major criterion (≥15 mast cells in aggregates in bone marrow or extracutaneous biopsies) and one of three minor criteria (>25% of mast cells are malformed, KIT mutation in codon 816, mast cell CD25 expression, or serum total tryptase >20 ng/mL) [1]. SM management depends on the subtype and extent of the disease, ranging from supportive symptom control to cytoreductive therapy. Further research aims to elucidate the molecular pathogenesis further and identify targeted therapies.

Indolent SM (ISM) is the most common SM variant, accounting for up to 70% of cases [2]. Patients with ISM are commonly asymptomatic but may experience sporadic skin lesions, pruritus, flushing, gastrointestinal complaints, headache, and fatigue [3]. Although ISM has a good prognosis relative to other SM subtypes, patients with ISM are at risk for anaphylaxis and may progress to advanced SM in about 3-4% of cases, resulting in poor outcomes and reduced life expectancy. Therefore, monitoring for disease progression is recommended [2]. The subset of SM cases that are asymptomatic and diagnosed incidentally is unknown. Identifying asymptomatic cases is crucial for appropriately monitoring disease progression and better managing symptoms when they eventually occur.

Gastrointestinal involvement occurs in over 80% of SM cases [4]. Endoscopic findings range from subtle mucosal changes to overt polyposis [5]. Thus, colonoscopy with biopsy is becoming a valuable supportive investigative tool, providing visual confirmation of gastrointestinal lesions and dense mast cell infiltrates in the colon mucosa. This is especially useful for diagnosing patients without cutaneous manifestations.

## Case presentation

**Figure 1:**
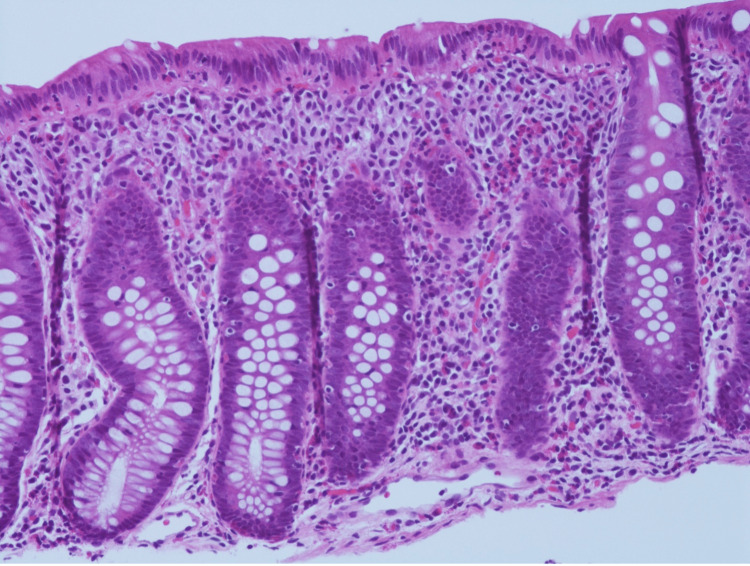
Hematoxylin and eosin (H&E) 20x. Right colon with increased eosinophils and mononuclear cells in the lamina propria.

A 66-year-old female with no significant past medical history underwent her first colonoscopy as a part of a routine colorectal cancer screening, which is recommended for individuals in her age group. The procedure was not prompted by any specific symptoms or conditions. The colonoscopy revealed three polyps in the cecum, which were resected. Upon hematoxylin and eosin (H&E) staining, aggregates of cells with a morphology suggestive of mast cells were present as subepithelial bands in the upper portion of the lamina propria in all three tissue fragments (Figure 1).

**Figure 2:**
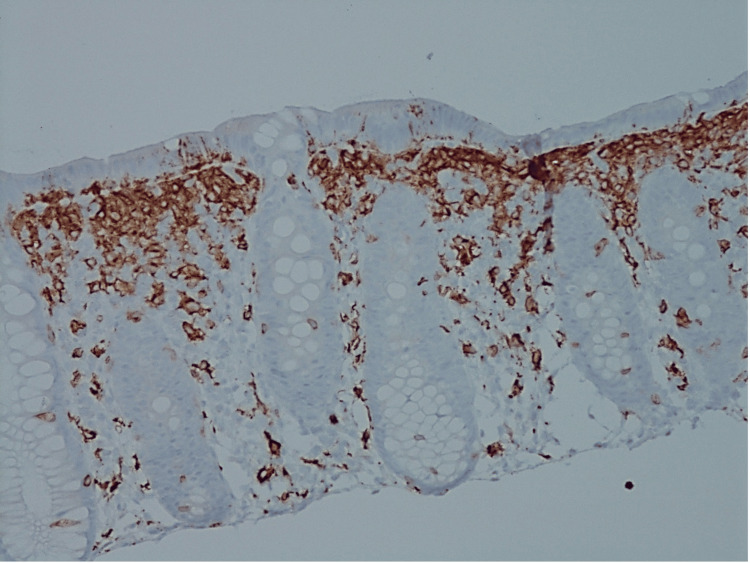
CD117 Immunohistochemistry (IHC) 20x. Note the subepithelial zone of mast cells, in addition to increased number in lamina propria.

To confirm the presence of mast cells, immunohistochemistry studies were performed. The suspected mast cells stained positive for CD117, a marker for mast cells and interstitial cells of Cajal, and CD25, which is aberrantly expressed in neoplastic mast cells (Figure 2). The cells were negative for CD1a, S100, and SOX10, markers for Langerhans cells, melanocytes, and neural crest-derived cells, respectively. This staining pattern confirmed the diagnosis of SM based on the WHO criteria of multifocal dense infiltrate of mast cells (more than 15 cells in aggregates) in an extracutaneous organ and aberrant CD25 expression by mast cells.

**Figure 3:**
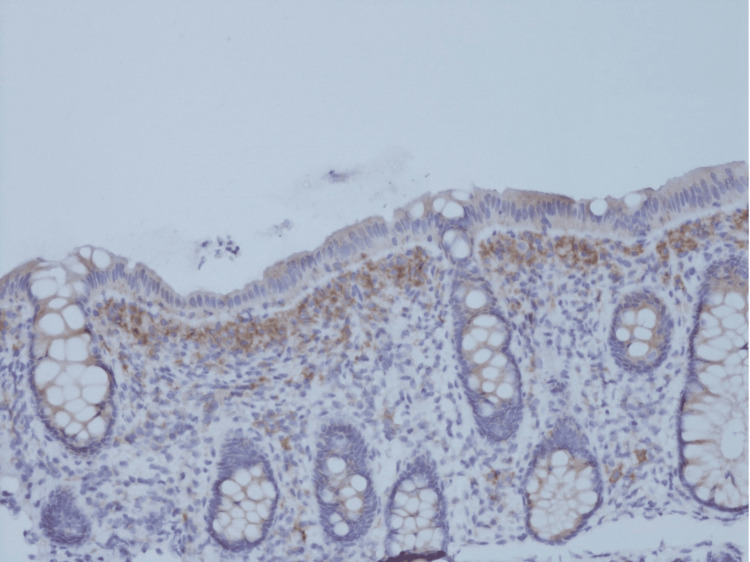
CD25 Immunohistochemistry (IHC) at 20x. Note the sub-epithelial zone of mast cells, a feature characteristic of gastrointestinal (GI) mucosal involvement by systemic mastocytosis.

To further characterize the extent of mast cell infiltration and rule out concomitant lymphoproliferative disorders, a repeat colonoscopy with twelve random colonic biopsies in both the ascending and sigmoid colon was performed. The biopsies were subjected to both histopathology and flow cytometry. The pathologists found confluent subepithelial bands of CD25+ mast cell infiltrate (Figure 3) with counts over 100/hpf at the right and left colon biopsy sites. The mast cells were negative for CD2, another aberrant marker in neoplastic mast cells. Flow cytometry ruled out lymphoproliferative disorders and showed a small population of mast cells with aberrant CD25 expression, further supporting the diagnosis of SM.

Additional laboratory tests, including methylhistamine, dinor-11 beta prostaglandin, serum tryptase, liver function tests, and complete blood count, were within normal limits except for a slightly elevated urinary leukotriene E4. Testing was negative for the KIT D816 mutation in peripheral blood.

The patient underwent a core bone marrow biopsy and aspirate to subtype the SM and rule out more aggressive forms. The bone marrow showed no mast cell disorder by immunohistochemistry and morphology, with the CD117 stain highlighting only a few singly scattered mast cells without any clusters. CD20 and CD3 highlighted scattered small B-cells and T-cells, respectively, and CD34 highlighted rare blasts, accounting for less than 1% of nucleated cells. These findings, along with the lack of symptoms and unremarkable abdominal and pelvic CT scans, led to the subtyping of the patient's SM as ISM.

Due to the lack of symptoms, starting antihistamines or mast cell stabilizers is currently unnecessary. However, the patient was advised to carry an epinephrine auto-injector as a precaution against possible anaphylactic reactions.

## Discussion

This case reveals an unexpected finding of isolated gastrointestinal (GI) mastocytosis without any associated skin manifestations or clinical symptoms of mast cell activation on routine screening colonoscopy. While prior literature describes GI involvement in over 80% of SM cases [4], patients invariably exhibit some degree of cutaneous lesions or symptoms, such as flushing, pruritus, or urticaria. However, this patient demonstrated multifocal mast cell infiltration on two colonoscopies with >100 mast cells/hpf at every random biopsy site confined to the colorectal mucosa despite being entirely asymptomatic. These findings challenge the assumption that SM with GI involvement is always symptomatic.

This case of truly occult disease emphasizes the importance of considering the diagnosis even without apparent symptoms. Up to half of SM patients may experience an anaphylactic reaction at some point, highlighting the need for heightened diagnostic awareness. In one study, severe episodes of anaphylaxis were more likely to be seen in patients with ISM or SSM subtypes compared to more aggressive forms [6]. Although anaphylactic reactions in asymptomatic SM patients have not been well-documented, clinicians should consider biopsy and histologic examination if routine procedures like colonoscopy reveal subtle abnormalities. This proactive approach helps prevent life-threatening anaphylaxis.

Pathologists' role in identifying SM in this asymptomatic patient was crucial. The recognition of subtle mast cell infiltrates on the initial H&E stain and the use of immunohistochemistry stains were instrumental in establishing the diagnosis. The positive staining for CD117 and the aberrant expression of CD25 confirmed the presence of neoplastic mast cells, while the negative staining for CD1a, S100, and SOX10 helped exclude other cell types that can mimic mast cells on H&E stain. This case underscores the importance of a thorough histopathological examination in routine endoscopic procedures, even in asymptomatic patients. The case also emphasizes the importance of a multidisciplinary approach, involving pathologists, gastroenterologists, and hematologists, in the diagnosis and management of SM.

## Conclusions

This case expands our understanding of the varied presentations of systemic mastocytosis and emphasizes the crucial role of pathologists in its diagnosis, especially in occult cases. It also underscores the need for a broader diagnostic perspective in GI assessments, even in asymptomatic patients. The findings emphasize the need for heightened vigilance, thorough histopathological examination, and a multidisciplinary approach to ensure timely diagnosis and appropriate management. Sharing this experience raises awareness about this rare condition, highlighting the importance of collaboration among healthcare professionals to improve patient outcomes and prevent potential complications, such as life-threatening anaphylaxis.
